# New Frontiers in the Pathophysiology of Hypertensive Pregnancy Disorders: A Systematic Review of Molecular Insights into Preeclampsia

**DOI:** 10.3390/cimb48030294

**Published:** 2026-03-10

**Authors:** Daiana Anne-Marie Constantin (Dimcea), Aida Petca

**Affiliations:** Department of Obstetrics and Gynecology, “Carol Davila” University of Medicine and Pharmacy, 050474 Bucharest, Romania; daiana-anne-marie.dimcea@drd.umfcd.ro

**Keywords:** molecular mechanisms, hypertensive disorders during pregnancy (HDP), preeclampsia (PE)

## Abstract

Background: Hypertensive disorders during pregnancy (HDP) pose a significant health risk to both mothers and infants. Despite extensive research, the precise pathophysiology of preeclampsia remains elusive, posing a formidable challenge to clinicians and researchers alike. This systematic review aims to summarize recent advances in understanding PE pathophysiology with particular emphasis on molecular mechanisms involved, as well as the roles and effects of genetics and hormones in PE. Methods: The literature was searched to identify clinical research investigating the underlying molecular and cellular mechanisms of PE, along with the genetic, hormonal, and pathophysiological changes. All studies were solicited from the databases PubMed, Scopus, and ScienceDirect according to the PRISMA 2020 workflow. The eligibility criteria were developed using the PECO (Population, Exposure, Comparator, Outcome) framework. Results: The most common molecular mechanisms found were: angiogenetic dysregulation and endothelial injury mechanism (*n* = 17), oxidative/redox imbalance and mitochondrial dysfunction mechanisms (*n* = 14), immune, inflammatory, and complement mechanisms (*n* = 13), trophoblast invasion, differentiation, and placental remodeling mechanism (*n* = 13), epigenetic and non-coding RNA regulation mechanisms (*n* = 9). Most of the included articles demonstrate more than one molecular mechanism, and the total number for each mechanism reflects the extent to which it is demonstrated in the studies. Conclusion: The complex development of hypertensive pregnancy disorders reflects numerous molecular mechanisms implicated in this process. From genetic and environmental influences on placental dysfunction to epigenetic changes and systemic dysregulation, each mechanism contributes to health outcomes for both the mother and the child.

## 1. Introduction

Hypertensive disorders during pregnancy (HDP), which include gestational hypertension, chronic hypertension, and preeclampsia (PE), pose significant health risks to both mothers and infants [1]. Recent estimates suggest that approximately 2 to 15% of all pregnancies are affected by these conditions, and the prevalence is expected to rise as maternal age increases and risk factors, such as obesity and metabolic syndrome, become more common [2,3].

From these conditions, preeclampsia (PE) affects 3–8% of all pregnancies. It is the leading cause of perinatal morbidity and mortality in developed countries, accounting for about 16–18% of maternal deaths and roughly 40% of fetal and neonatal deaths [4]. The International Society for the Study of Hypertension in Pregnancy (ISSHP) defines PE as the onset of hypertension after 20 weeks’ gestation accompanied by proteinuria or evidence of maternal acute kidney injury, liver dysfunction, neurological symptoms, hemolysis, thrombocytopenia, or fetal growth restriction (FGR). There are two types of PE: early-onset disease, which develops before 34 weeks’ gestation, and late-onset disease, which develops after 34 weeks’ gestation [5,6].

Knowledge of PE pathophysiology is still evolving. PE represents a complex syndrome with several potential pathways leading to disease development, in which mechanisms involved in early-onset PE differ from those implicated in late-onset PE [7].

Preeclampsia diagnosed before 34 weeks of gestation has been associated with inadequate trophoblast invasion, resulting in a pronounced maternal inflammatory response [8,9,10]. Immunological disturbances are frequently observed in this context, including altered profiles of T helper (TH) lymphocytes and elevated levels of CD19+, CD5+ B lymphocytes, which collectively substantiate the preeclampsia (PE) phenotype [11]. On the other hand, late-onset preeclampsia may result from a maternal predisposition to cardiovascular pathology. In this condition, the diseased placenta releases factors that cause widespread endothelial damage in maternal organs, such as the kidneys, liver, and nervous system [12].

Despite extensive research, the precise pathophysiology of preeclampsia remains elusive, posing a formidable challenge to clinicians and researchers alike. Although the exact molecular pathways have not been fully elucidated, new insights in this field are essential, as they are believed to play a pivotal role in identifying potential targets for future intervention and prevention. These advances carry significant implications for both maternal and offspring health outcomes.

In this manner, this systematic review aims to summarize recent advances in understanding PE pathophysiology with particular emphasis on molecular mechanisms involved, as well as the roles and effects of genetics and hormones in PE.

## 2. Materials and Methods

**Figure 1:**
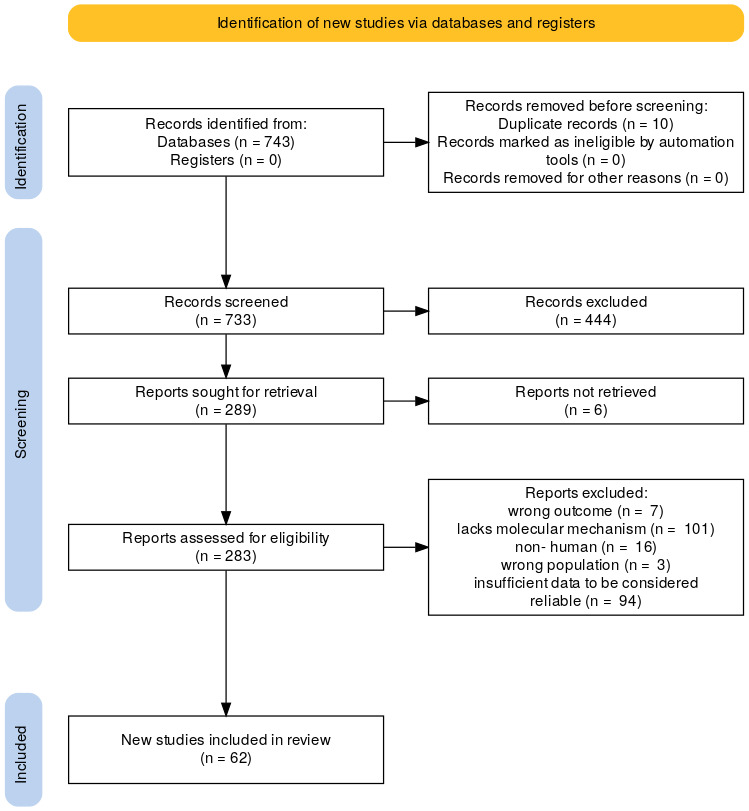
Flowchart of database searching, screening, and the inclusion of the references that were collected from the literature according to the PRISMA 2020 workflow.

The literature was searched to identify clinical research investigating the underlying molecular and cellular mechanisms of PE, along with the genetic, hormonal, and pathophysiological changes. Figure 1 illustrates the flowchart for database searching, screening, and reference inclusion, following the PRISMA 2020 workflow. The protocol for this systematic review was registered with PROSPERO (ID: CRD420261299381).

In the current systematic review, all studies were solicited from the databases PubMed, Scopus, and ScienceDirect using the search terms’ preeclampsia’, ‘gestational hypertension’, ‘pregnancy’, and ‘molecular mechanisms’ (Supplementary File S1 records the exact queries used in each database, with filters and the search date, for PRISMA reproducibility). For screening and selection in the second stage, only English-language full-text articles were considered for inclusion. In the next stage, duplicated articles were excluded. Articles published before 2015 were also excluded to ensure the systematic review’s novelty. From a total of 743 articles identified in the screening process, 62 articles met the criteria for further inclusion [13,14,15,16,17,18,19,20,21,22,23,24,25,26,27,28,29,30,31,32,33,34,35,36,37,38,39,40,41,42,43,44,45,46,47,48,49,50,51,52,53,54,55,56,57,58,59,60,61,62,63,64,65,66,67,68,69,70,71,72,73,74].

The eligibility criteria were developed using the PECO (Population, Exposure, Comparator, Outcome) framework:

- Population: human studies of pregnant women diagnosed with HDP: PE, gestational hypertension, or eclampsia
- Exposure/Focus: investigations of molecular or cellular mechanisms implicated in HDP pathogenesis, including (but not limited to) oxidative stress, angiogenic/antiangiogenic signalling, gene/protein expression, epigenetic regulation (DNA methylation, histone modification, non-coding RNA), immune-inflammatory pathways, endothelial and placental dysfunction, and signal transduction
- Comparators: normotensive pregnancy controls or internal comparators
- Outcomes: mechanistic molecular endpoints
- Study designs: randomized controlled trials (RCTs) and non-randomized human studies (quasi-experimental or mechanistic observational designs, such as cohort, case-control, or cross-sectional) that report molecular endpoints
- Limits: publication window (1 January–1 September 2025), English language, peer-reviewed journal articles, full-text available.

To strengthen clinical interpretability and reduce avoidable heterogeneity, we applied three a priori restrictions. First, we limited inclusion to human pregnancy studies, because placentation, spiral-artery remodeling, immune tolerance and angiogenic signalling show important inter-species differences. Second, we restricted to English-language full texts to ensure accurate data extraction and consistent risk-of-bias appraisal within available resources. Third, we included RCTs and mechanistic observational designs (cohort, case-control, cross-sectional and quasi-experimental studies) because these designs permit structured comparison against normotensive controls or internal comparators and allow appraisal using established risk-of-bias tools. Finally, we excluded studies reporting isolated biomarker differences without pathway context or mechanistic testing because the review objective was to synthesize pathophysiological mechanisms, not biomarker associations alone.

The exclusion criteria are represented by:

- Animal or in vitro-only studies
- Purely clinical/diagnostic/prognostic epidemiologic papers without mechanistic molecular investigation
- Reviews, editorials, commentaries, protocols, conference abstracts, and case reports/series
- Non-English or outside the date window articles
- Full text not accessible
- Studies that only report biomarker levels without pathway context or mechanistic testing
- Studies with unclear ethics or serious methodological deficiencies preclude appraisal.

Subsequently, two experts independently reviewed each article’s content, including the research design and materials, and identified studies suitable for inclusion. Any discrepancies between the reviewers were discussed until a consensus was reached.

Two reviewers independently extracted data using a piloted form. Items included: study design, main molecular mechanism, detailed mechanism, timing of sampling (gestational age, labour status), tissue/source (placenta, trophoblast), endothelium, serum/plasma), comparator(s), molecular endpoints and effect metrics, assay platform and validity (qPCR/RNA-seq/ELISA/Western blot). Discrepancies were reconciled by discussion.

Regarding risk of bias (RoB), the reviewers independently assessed each article, while discrepancies were revised through discussion. For RCTs, we applied Cochrane RoB 2.0 across five domains: randomization process, deviations from intended interventions; missing outcome data; measurement of the outcome (including laboratory blinding and assay validity); and selection of the reported result. For observational studies, we used the Newcastle-Ottawa Scale (NOS) (cohort or case-control as appropriate), converting total stars (0–9) to overall RoB: 7–9 = Low risk (Good), 5–6 = Some concerns (Fair), 0–4 = High risk (Poor). Mechanism-specific appraisal emphasized assay validity/reproducibility, predefined analytic thresholds, and appropriate handling of batch effects and multiplicity. Study-level RoB judgments (Low/Some concerns/High) were derived from domain ratings and summarized narratively and in tables.

Finally, 62 articles were identified for review from the 289 articles solicited in the initial search.

Considering the anticipated variability across tissues, platforms, and mechanistic endpoints, we organized a structured narrative synthesis by the primary mechanism analysed in the included studies. We emphasized results validated by reliable assays, proper normalization and blinding procedures, and controls for multiple comparisons.

## 3. Results

Across the included articles, the most common molecular mechanisms found were: angiogenetic dysregulation and endothelial injury mechanism (*n* = 17), oxidative/redox imbalance and mitochondrial dysfunction mechanisms (*n* = 14), immune, inflammatory, and complement mechanisms (*n* = 13), trophoblast invasion, differentiation, and placental remodeling mechanism (*n* = 13), epigenetic and non-coding RNA regulation mechanisms (*n* = 9). Most of the included articles demonstrate more than one molecular mechanism, and the total number for each mechanism reflects the extent to which it is demonstrated in the studies (Table 1). Supplementary File S2 records all the articles mentioned in Section 3.

All eligible studies contributed to the mechanism-specific counts and RoB summaries. The cited studies were selected a priori as representative and methodologically informative: we prioritized articles in which the main mechanism was completely demonstrated and used secondary-coded studies only when needed to illustrate cross-mechanism links; we preferentially chose studies with clear mechanistic support (e.g., coherent pathways, mapping and/or functional validation), a better methodological quality (lower RoB) and more recent published articles.

### 3.1. Angiogenetic Dysregulation and Endothelial Injury Mechanism

In our systematic review, we found that 17 of the included articles described angiogenic dysregulation and endothelial injury as a primary or secondary mechanism. Among these, 13 studies were primary angiogenic/endothelial studies, while the other 4 articles displayed angiogenic features secondarily. From the primary studies, overall RoB was Low in 23% (3 articles) and in 77% (10 articles) showed some concerns, but there were no High-risk studies. The secondary group demonstrated a similar profile: low in one article (25%) and some concerns in 3 (75%), with no study judged as High risk.

From the articles in the primary set, it was demonstrated that pro- and antiangiogenic factors are central to the pathophysiology and diagnosis of PE. Antiangiogenic factors, such as soluble fms-like tyrosine kinase-1 (sFLT-1) and soluble Endoglin (sEng), are found to be elevated in maternal circulation before the clinical features of PE appear [13]. Overexpression of sFLT-1, a soluble protein elevated in PE, can induce vascular sensitivity to vasopressors, such as Angiotensin II (Ang II). sFLT-1 acts on the maternal endothelium to inhibit endothelial nitric oxide synthase (eNOS) phosphorylation, which is associated with increased blood pressure, increased vessel oxidative stress, and increased sensitivity to vasopressors [14] (Table 2).

In a study conducted by Wang Y. et al., it was reported that high temperature requirement A4 (HtrA4) is a placenta-specific protease that is significantly increased in the circulation of early-onset PE patients. At high concentrations, HtrA4 disrupted tube formation in ombilical vein endothelial cells (HUVECs), disturbed cellular integrity, and increased cellular permeability, suggesting that this may contribute to endothelial dysfunction and the development of early-onset PE [15].

On the other side, an important role in the prevention of PE that acts through angiogenetic mechanisms comes from the inhibitor of DNA binding family protein (ID), which is a class of transcription factors and suppressor of mothers against decapentaplegic (SMAD) family proteins that play an important role in the proliferation, migration, angiogenesis, and permeability of vascular endothelial cells. Alpha-1-antitrypsin knockdown altered the expression of SMAD family and ID family genes, and further demonstrated that it positively regulated ID4 expression through activating SMAD2, protecting cells from hypoxia injury, relieving preeclampsia symptoms, and finally contributed to the prevention of PE progression [16].

PE is a systemic vascular disorder and is associated with increased sensitivity to Ang II and hypertension. To test the hypothesis that regulator of G protein signaling 5 (RGS5) is involved in pregnancy-related vascular adaptation, myometrial arteries from women with normal or hypertensive/pre-eclamptic pregnancies were analyzed. RGS5 expression was significantly (*p* = 0.0015) reduced in hypertensive/pre-eclamptic pregnancies, suggesting that low RGS5 levels contributed to the vascular pathophysiology of PE. The study demonstrated that peroxisome proliferator-activated receptor (PPAR) agonist treatment for RGS5-related hemodynamic maladaptations may be effective during the second half of pregnancy at the peak of volume expansion [17]. Together, these mechanistically anchored studies indicate that antiangiogenic load and endothelial injury are not merely correlative: they are actionable nodes where pathway-guided interventions can restore function.

In the secondary set of included articles, angiogenic and endothelial phenotypes appear as downstream readouts of other primary programs, but still reveal important directions with translational promise. Insufficient invasion of trophoblast cells into the uterine decidua is associated with PE. G protein-coupled estrogen receptor (GPER) is expressed in human trophoblast cells, and downregulated levels are noted in PE. Estrogen/GPER signaling promoted trophoblast invasion and pro-angiogenetic behavior via Yes-associated protein (YAP) -mediated angiopoietin-like 4 (ANGPTL4), with receptor blockade and gene knockdown abrogating the effect on the endocrine-to-angiogenic bridge [18].

Gut microbiota imbalance represents a novel and recent discovery in the pathogenesis of PE. In the study conducted by Wang J. et al. on 28 patients with PE and 39 controls, which was published in 2024, the results showed that the level of trimethylamine N-oxide (TMAO) and the abundance of its source bacteria had significantly increased in patients with PE, and were positively correlated with clinical progression. Mechanistically, TMAO promotes progression of PE by regulating inflammatory and oxidative stress-related signaling pathways, affecting the migration and angiogenesis of vascular endothelial cells [19].

At the cytokine level, proteomics analysis reveals that interleukin 6 (IL-6) regulates trophoblast function by interacting with multiple proteins and pathways. It was found that IL-6 levels were higher in the placenta of the PE group (*n* = 12) than in the normal group (*n* = 9). The difference between the two groups was statistically significant (1.47  ±  1.30 vs. 3.60  ±  1.37, *p*  =  0.018). IL-6 suppressed the proliferation and invasion of HTR-8/SVneo cells but promoted the angiogenesis of HUVECs [20].

Complementing these pathways, the transcription factor peroxisome proliferator-activated receptor-γ (PPARγ) promotes healthy trophoblast differentiation, but is dysregulated in the pre-eclamptic placentas. Grimaldi B. et al. evaluated placental PPARγ activation in placental tissue/ex vivo experiments (study recruitment 2013–2017). PPARγ protein expression was significantly reduced in preeclamptic placentas compared with controls (0.47 ± 0.13 vs. 1.03 ± 0.11 relative units; *p* = 0.01; *n* = 7). Pharmacologic PPARγ activation increased Heme oxygenase-1 (HO-1) and shifted the placental angiogenic stress signature toward a more balanced profile [21].

From this point of view, these data show that even when angiogenic imbalance is secondary, endothelial dysfunction remains a common point of convergence- and a rational target-across diverse upstream mechanisms.

In synthesis, the primary angiogenic/endothelial corpus identifies core injury pathways, such as sFLT1/FLT1-dominant antiangiogenic signaling, HtrA4-driven endothelial damage, and protective AAT/Smad2-ID4, while the secondary corpus situates hormone receptors, cytokine signaling (IL-6), and nuclear receptor programs (PPARy-HO-1), which contribute secondarily to stimulate angiogenetic dysregulation. These insights motivate mechanism-guided trials that explicitly track endothelial function and angiogenic balance as primary endpoints.

### 3.2. Epigenetic and Non-Coding RNA Regulation Mechanisms

Records were classified as primary when the main mechanism explicitly recorded epigenetic or non-coding RNA (ncRNA) regulation, and secondary when such regulation appeared along with other mechanisms, following the same rules as in previously described molecular mechanisms.

Across all the included articles that mentioned epigenetic/ncRNA features in any field (*n* = 9), 5 records were primary, and 4 were secondary. In the primary subset, overall risk of bias (RoB) was Low in 2 (40%), Some concerns in 3 (60%), and High in none (0%). In the secondary subset, RoB was Low in 0 (0%), Some concerns in 4 (100%), and High in 0. The dominant limitations were incomplete blinding, laboratory assessments, heterogeneous normalization/multiplicity in omics, and partial control of gestational age and clinical confounding features (Table 2).

An imprinting-focused epigenetic study evaluated Pleckstrin homology-like domain family A member 2 (PHLDA2) expression and promoter methylation in third-trimester placental tissue from pregnancies with PE and from normotensive controls. In PE placentas, PHLDA2 mRNA and protein levels were significantly higher than in controls (*p* = 0.001). This increase in expression was accompanied by hypomethylation of the PHLDA2 promoter region, consistent with epigenetic de-repression at this imprinted locus. The authors interpreted the PHLDA2 hypomethylation-overexpression pattern as a potential contributor to PE pathophysiology and discussed its possible relevance for future diagnostic/therapeutic exploration [22].

A placental long non-coding RNA (IncRNA)-centered transcriptomic network was demonstrated to be associated with novel early-onset PE. The upregulation of differentially expressed long non-coding RNA (DElncRNAs), such as RP11-211G3.3 and RP11-65J21.3, was validated in clinical placenta samples from patients with early-onset PE by quantitative reverse transcription PCR, which increased trophoblast proliferation, directly linking non-coding hubs to angiogenic control [23]. At the epitranscriptomic layer, upregulated methyltransferase-like 3 (METTL3) and m^6^A levels and downregulated transmembrane BAX inhibitor motif containing 6 (TMBIM6) were observed in PE placentas under endoplasmic reticulum (ER) stress. Moreover, the knockdown of METTL3 had a beneficial effect on human trophoblast row (HTR-8)/SVneo cells under ER stress, as it decreased the levels of methylated TMBIM6 mRNA. The overexpression of TMBIM6 was beneficial to HTR-8/SVneo cells under ER, as it could neutralize the harmful effect of METTL3 overexpression. In conclusion, the METTL3/TMBIM6 axis exerts a significant role in trophoblast dysfunction resulting in PE, while inhibiting METTL3 may provide a novel therapeutic approach for PE [24].

Finally, imprinting drift extended to multiple-locus changes with oxidative DNA damage markers and endocrine correlations (IGF2 and cortisol reductions), demonstrating how epigenetic instability links metabolic and hormonal signals in the pre-eclamptic placenta [25]. Across these studies, epigenetic and ncRNA controls appear as upstream regulators that adjust trophoblast cell-cycle and apoptosis, syncytialization processes, and angiogenic balance.

In studies where epigenetic/ncRNA signals were not the primary coding mechanism, these regulators nonetheless acted as cross-cutting drivers of immune activation, endothelial injury, and adhesion/signaling defects. MicroRNA-223-3p (miR-223-3p) plays an important role in regulating monocyte-macrophage differentiation and the pro-inflammatory response, and is localized in trophoblast cells of the placenta. In a case-control study of 60 pregnant women from Jiangsu Province (30 preeclampsia; 30 controls), placental NOD-like receptor family pyrin domain containing 3 (NLRP3) mRNA expression was significantly higher in the PE group than in the normotensive pregnancies (*p* < 0.001) [26]. An angioneogenesis-focused study identified a placental lncRNA (MG828507) located upstream of FLT1, suggesting a cis-regulatory relationship that links ncRNA regulation to sFLT1/anti-angiogenic signaling [27]. Two studies of syncytiotrophoblast stress promote Syncytiotrophoblast Membrane Extracellular Vesicles (STB-EV) release into the maternal circulation and are associated with increased production of 5′-tRNA fragments (5′-tRFs). Within the EV-associated 5′-tRF repertoire, 5′-prime tRNA-derived fragmentoriginating from Glutamic acid with CTC anticodon (5′-tRF-Glu-CTC) was reported as the most abundant PE-upregulated fragment [28]. In a second STB-EV study, PE-derived EVs enriched with neutrophil extracellular traps (NETs) and DNA/RNA cargo were shown to propagate endothelial dysfunction, including ROS generation and junctional disruption [29].

This mechanism-focused synthesis indicates that epigenetic and ncRNA regulation is both a primary disease program-modulating trophoblast cell-cycle, ER-stress/apoptosis, and angiogenic signaling, and a cross-mechanism amplifier of immune, metabolic, and adhesion pathways.

### 3.3. Oxidative/Redox Imbalance and Mitochondrial Dysfunction Mechanisms

Across all included records in which oxidative/redox or mitochondrial mechanisms were coded in any field (*n* = 14), 10 studies were primary, and 4 were secondary. Most experiments used placental tissue and/or trophoblast models (12/14; 85.7%), with a smaller subset focusing mainly on maternal blood or leukocytes (2/14; 14.3%) or on other mixed compartments. Late-pregnancy or delivery sampling predominated (13/14; 92.8%), whereas only 1/14 (7.2%) incorporated the first trimester (Table 2).

The overall RoB was Low in 2 studies (14.2%), some concerns in 12 studies (85.8%), and High in none (0%). In the primary oxidative/mitochondrial group (*n* = 10), RoB was Low in 2 (20%), some concerns in 8 (80%), and High in 0 (0%). In the secondary group (*n* = 4), RoB was Low in 0 (0%), Some concerns in 4 (100%), and High in 0 (0%). The main limits of the included articles were incomplete blinding and cross-sectional designs with modest sample sizes.

Primary oxidative/mitochondrial studies converge on a model in which disturbed ROS handling, ferroptosis, and impaired mitochondrial bioenergetics jointly constrain trophoblast function and vascular homeostasis. A large case-control study used high-throughput expression profiling to identify oxidative-stress-related hubs, including nicotinamide adenine dinucleotide phosphate (NADPH)- oxidase and antioxidant enzymes that discriminated PE from the normotensive group, nominating them as candidate circulating biomarkers and upstream regulatory nodes in PE pathogenesis [30].

Hypoxia-induced reactive oxygen species (ROS) accumulation causes chronic trophoblast injury and contributes to PE. Glutathione S-transferase P1 (GSTP1) is a major regulator of ROS; however, it remains unknown whether GSTP1 is involved in ROS regulation under hypoxic conditions. In a study published in 2023, GSTP1 expression in first-trimester villi placentas was much higher than in full-term placentas (*p* < 0.05). These data show that hypoxia-induced GSTP1 expression facilitates trophoblast cell proliferation, migration, and invasion and exerts a protective effect under hypoxic conditions, which may play an important role during the increase in PE [31]. Peroxiredoxin-1 (PRDX1) was similarly protective: PRDX1 knockdown in trophoblasts reduced autophagy, increased ROS, and impaired migration, whereas PRDX1 overexpression reversed the effect, indicating that the loss of PRDX1-dependent detoxification can couple oxidative stress to defective autophagy and trophoblast dysfunction [32].

Evidence for an iron-dependent ferroptosis component in PE has been reported at both the maternal immune-cell level and in placental tissue [33,34]. Lekva T. et al. investigated leukocytes and EVs from pregnancies with PE and controls and reported increased ferroptosis-related changes in maternal leukocytes [33]. In the studym reduction in anti-ferroptotic markers and selected lncRNA/mRNA signals were observed in leukocytes, whereas the same pattern was not consistently detected in EVs [33]. The authors further reported that these anti-ferroptotic reductions were not associated with maternal disease activity or plasma oxidative stress status [33]. Instead, the leukocyte profile was interpreted as being linked to attenuated anti-inflammatory expression in these cells [33]. At the placental level, a separate study linked solute carrier family 7-member 11 (SLC7A11)—dependent ferroptosis signaling to pannexin-1 (PANX1) [(AUC = 0.997) (95%CI: 0.954–1.000)] and toll-like receptor 4 (TLR4) [(AUC = 0.988) (95%CI: 0.938–1.000)] expression in women with PE [34]. Based on these associations, PANX1/TLR4 were proposed as upstream regulators and potential diagnostic markers of ferroptotic stress in the placenta [34].

In studies where oxidative/mitochondrial features were coded only as secondary mechanisms, redox imbalance frequently mediated the interface between immune activation, complement, EV, metabolic signals, and endothelial injury.

Many pathological NETs at the maternal–fetal interface are believed to be among the main pathogenic factors leading to PE. A recent placental study of NETs demonstrated that they can cause trophoblast HMGB1-mediated functional damage during the occurrence and development of PE. High mobility group box 1 (HMGB1) produces a marked effect in the PE cascade of oxidative stress involving NETs [35]. In a complementary study published in 2024, circulating EVs and NETs from participants with PE induced endothelial activation, ROS generation, and barrier disruption, positioning NET-decorated EVs as carriers of oxidative and inflammatory cargo that propagate vascular dysfunction [29].

Overall, the secondary literature suggests that oxidative/mitochondrial stress is often the biochemical ‘currency’ through which immune, complement, metabolic, and EVs pathways exert their damaging effects.

### 3.4. Immune, Inflammatory and Complement Mechanisms

The total number of the included articles in which immune, inflammatory, or complement features were 13:7 studies were primary, and 6 were secondary. Most of the experiments used placental tissue (often with trophoblast): 11 studies (84.6%) were placenta-only and 2 (15.4%) combined placenta with maternal blood or immune cells. Sampling was mainly from late gestation: 10/13 (76.9%) were based on late-pregnancy or delivery/postpartum specimens, while only 3/13 studies (23.1%) incorporated first-trimester material (Table 2).

The overall RoB was Low in 3 studies (23%), Some concerns in 10 (77%), and High in 0 study (0%). In the primary immune/inflammatory group (*n* = 7), RoB was Low in one study (14.2%), some concerns in 6 (85.8%), without any High articles found (0%). In the secondary group (*n* = 6), RoB was Low in 2 (33.3%), some concerns in 4 (66.7%), and High in none (0%). As in previous mechanisms, most downgrades were cross-sectional designs with modest sample sizes, and incompatibility blinding or single-centre cohorts.

Altered Natural Killer (NK)-trophoblast immune-recognition signals in PE were supported by two relevant mechanistic studies evaluating the placental transcripts/proteins linked to cytotoxic immune activation [36,37]. In the study by Ito M. et al., the Nectin cell adhesion molecule 4 (NECTIN-4) transcript levels were significantly elevated in preeclamptic placentas compared with those of uncomplicated pregnancies (*p* < 0.0001) [36]. In a separate study, expression of endoplasmic reticulum aminopeptidase 1 and 2 (ERAP1/ERAP2) was significantly lower in women with PE than in normotensive controls (*p* < 0.05) [37]. In the same report, ERAP2 protein expression in normotensive pregnancies at delivery was unchanged compared with the first trimester [37]. Reduced ERAP1/ERAP2 expression was associated with increased activated NK and T cells and increased cytokine release, consistent with a link between impaired antigen processing and exaggerated cytotoxic immune responses at the maternal–fetal interface [37]. Together, increased NECTIN4 transcripts reduced ERAP1/ERAP2 expression, providing convergent evidence for altered placental immune recognition pathways in PE [36,37].

An important mechanistic cluster in the included literature concerns sterile inflammation driven by cholesterol crystal-associated inflammasome signaling at the maternal–fetal interface in PE [38,39]. In decidual explant experiments, cholesterol crystals triggered NLRP3-dependent IL-1β release, and PE samples showed stronger NLRP3/IL-1β signals together with increased crystal deposition compared with controls [38]. Consistent with heightened inflammasome-linked inflammation, another included study reported significantly higher endogenous expression of NLRP1, NLRP3, and High mobility group box protein (HMGB1) in PE than in controls, with further increases in cultures treated with H_2_O_2_ (*p* < 0.05) [39]. Upstream inflammatory regulation was also supported by evidence implicating nuclear factor kappa B (NF-kB)—centered signaling in PE placental [40]. In that study, Ubiquitin-specific protease 14 (USP14) and pro-inflammatory cytokines were upregulated in placental tissue from PE participants, and inhibition of USP14 significantly attenuated hypoxia/reoxygenation-induced Nf-kB activation and cytokine production [40]. Together, these data support a model in which sterile triggers and oxidative stress amplify placental inflammation through convergent inflammasome-linked and NF-kB-regulated pathways [38,39,40].

Complement regulation defects were directly implicated in several primary studies. The distribution and expression of Factor H (FH), which is a negative regulator of complement activation, were investigated in placental tissues and various placental cells from normotensive or PE women. FH was found to be considerably less expressed in the placental tissue of PE patients, resulting in complement over-activation with increased Complement component C3b and C5b-9 deposition [41]. Placental and systemic analyses of C3a receptor (C3AR1) identified altered expression and signalling in PE, suggesting that C3AR1 modulates inflammatory and angiogenic responses downstream of complement activation [42].

The primary studies support a model in which skewed macrophage/NK-cell responses, NLRP3 inflammasome activation, and complement dysregulation converge to drive inflammation that damages trophoblasts and maternal endothelium.

In studies where immune, inflammatory, or complement features were secondary, these pathways often mediated or modulated other primary mechanisms (trophoblast senescence, angiogenic signalling, epigenetic regulation).

Increasing evidence suggests that an exaggerated maternal systemic inflammatory response may play a central role in the pathogenesis of PE. Considering the growing evidence on microRNAs (miRNAs) and tissue-specific regulators of gene expression, it has been demonstrated in a recent clinical study of regulatory T cells conducted on PE participants (*n* = 29 patients with late-onset PE) an association with increased miR-210 and decreased forkhead box p3 (Foxp3), reduced circulating Treg frequency, and a shift to a pro-inflammatory profile (overexpression of IL-6, IL-16 with down expression of IL-10) [43]. Another study found reduced miR-144-3p in PE placentas. miR-144-3p directly targeted cyclooxygenase (COX-2), and its overexpression reduced COX-2 proteins, associating ncRNA dysregulation with prostaglandin-driven inflammation [44].

Several secondary studies situate inflammatory mechanisms within broader cell-death and stress programs. qPCR and Western blot analysis revealed in one recently published study that miR-135 was down-regulated while proprotein convertase subtilisin/Kexin-6 (PCSK6) and NLR pyrin domain containing 3 (NLRP3) inflammasome were up-regulated in placenta tissue in PE patients, leading to increased IL-1beta and Tumor Necrosis Factor (TNF)-alpha and reduced trophoblast viability, while miR-135 overexpression or NLRP3 inhibition attenuated these inflammatory anomalies [45]. The role of the miR-494/longevity protein Sirtuin 1 (SIRT1) axis in PE has been associated with placental senescence. SIRT1 expression was lower (*p* < 0.01), and miR-494 expression was higher (*p* < 0.001) in severe PE women (*n* = 20), this interaction playing a role in the mechanism of premature placental aging [46].

In conclusion, the secondary articles reinforce the idea that immune, inflammatory, and complement pathways are rarely isolated: they frequently serve as the biochemical interface through which epigenetic alterations, oxidative damage, and metabolic shifts manifest as vasculo-placental injury.

### 3.5. Trophoblast Invasion, Differentiation and Placental Remodeling Mechanism

Across all included articles in which any mechanism field referenced trophoblast invasion, differentiation, or placental remodeling (*n* = 13), 7 studies were primary, and 6 were secondary. Placental tissue was analysed in 11/13 (84.6%) and trophoblast cell models in 11/13 (84.6%); 2/13 (15.3%) also included maternal and fetal blood/plasma. The vast majority sampled at delivery or in late pregnancy: 9/13 (69.2%) were clearly late-gestation/delivery studies and 4/13 (30.8%) focused on early gestation.

Overall RoB for the trophoblast/remodeling subset was Low in 2 (15.3%), Some concerns in 11 (84.7%), and High in 0 (0%). Among primary studies (*n* = 9), RoB was Low in 1 (14.2%), Some concerns in 6 (85.8%), and High in 0 (0%). In the secondary set (*n* = 6), RoB was Low in 1 (16.6%), Some concerns in 5 (83.4%), and High in 0 (0%). Most downgrades reflected single-centre, cross-sectional designs with moderate to low sample sizes, imperfect adjustments for gestational age/severity, and heavy reliance on trophoblast cell lines (Table 2).

Primary trophoblast/placental remodeling studies converge on 3 linked axes: integrin and metalloprotease control of invasion and extracellular matrix (ECM) remodeling, epithelial-mesenchymal transition (EMT) and motility programs driven by non-coding RNAs and Notch, and thirdly, microenvironmental remodeling of the placental ECM.

Two related studies highlighted proteases that orchestrate placental ECM and syncytial architecture. Immunostaining of ADAM-like Decysin-1 (ADAMDEC1) in PE placentas was found to be weaker than that in the normal placenta, but human cytotrophoblast (CTB) marker CK7 had the opposite expression pattern. ADAMDEC1 protein was downregulated while thrombospondin-1 (THBS1) was upregulated; simultaneously, the expression of syncytialization markers Syncytin1 and beta-hCG was reduced in 6 PE placentas. These results implied that low expression of ADAMDEC1 might contribute to the insufficient differentiation of placental STBs and the development of PE [47]. In a second study, recombinant ADAMTS13 stimulated proliferation, migration, invasion, and network formation of trophoblastic cells in culture, while ADAMTS13 knockdown attenuated tube formation in HTR-8/SVNEO cells and reduced extravillous trophoblast outgrowth in placental explants [48].

Consistent with a primary role for trophoblast differentiation programs, another study investigated claudin-1 (CLDN1) in early-onset PE and reported CLDN1 downregulation with an EMT-like phenotype, suppressed Platelet Endothelial Cell Adhesion Molecule 1 (PECAM1) and IL-1β, impaired trophoblast invasion, and altered endothelial interaction. CLDN1 overexpression restored invasive capacity, supporting CLDN1 as a tight-junction regulator of EVT differentiation and vessel remodeling [49].

NcRNA studies reinforce EMT-based control of trophoblast invasion. MiR-3935 expression is significantly decreased in both placentas and peripheral blood specimens from PE patients, and functionally, miR-3935 promotes EMT of trophoblast cells [50]. In another report, Vimentin Antisense RNA 1 (VIM-AS1) was downregulated in hypoxia-induced EMT; VIM-AS1 suppression inhibited EMT and reduced trophoblast migration/invasion, while VIM-AS1 overexpression restored EMT and motility, indicating that hypoxia-sensitive lncRNAs can integrate oxygen tension with invasive programming [51].

**Figure 2:**
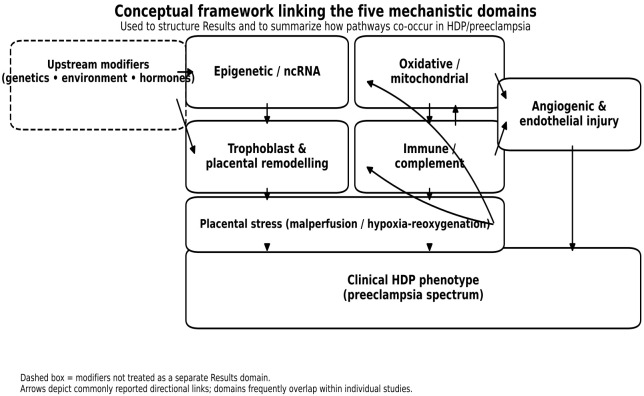
Conceptual framework linking the five mechanistic domains in HDP/PE occurrence.

In most secondary studies, trophoblast invasion and placental remodeling appeared as downstream or intermediate phenotypes embedded within broader epigenetic, immune, oxidative, metabolic, or angiogenic frameworks. This pattern is consistent with the conceptual framework in Figure 2, in which trophoblast behaviour acts as a shared effector pathway for multiple upstream stress signals.

Epigenetic regulation of trophoblast invasion was exemplified by a Histone deacetylases (HDAC9)- tissue inhibitor of metalloproteinases 3 (TIMP3) axis reported in the preeclampsia placenta. In this study, HDAC9 expression was reduced in PE placentas compared with normal placentas. Mechanistically, HDAC9 promoted trophoblast migration and invasion by repressing TIMP3, consistent with promoter histone hypoacetylation-mediated transcriptional control [52]. These findings support an epigenetic model in which HDAC9-dependent chromatin regulation tunes matrix remodeling and extravillous trophoblast motility through TIMP3 [52].

Beyond the placenta proper, one study reported that DNA Damage Inducible Transcript 4 (DDIT4) was downregulated in decidua; DDIT4 knockdown activated mTOR, reduced autophagy, and increased decidual apoptosis, impairing implantation-related remodeling, suggesting that early decidual stress contributes to later shallow placentation [53].

Endocrine-stress interactions were also linked to trophoblast invasion. In a study evaluating cytochrome P450 family 11 subfamily A member 1 (CYP11A1) expression in the placentation process, the results suggested that high expression induces trophoblast autophagy and inhibits trophoblastic invasion, which is associated with the etiology of PE; androgen receptor blockade partly reversed these effects, implicating endocrine-autophagy-matrix interactions in invasion defects [54].

Two secondary epigenetic studies emphasized imprinted genes and lncRNA networks in trophoblast and placental development. A paper published by Chen Y. et al. in 2024 built a placental lncRNA-mRNA network for early-onset PE, identifying lncRNAs linked to vascular endothelial growth factor A (VEGFA/FLT1) and trophoblast developmental pathways [23]. In another study in which imprinted genes were considered to play an important role in placentation and pregnancy, methylation and expression of potassium voltage-gated channel subfamily Q member 1, GNAS complex locus, mesoderm-specific transcript, and IGF2 were significantly altered in PE patients [25].

Finally, although coded as immune or metabolic as the main mechanistic pattern, some studies identified explicit trophoblast-invasion consequences, exemplified by the C3AR1 study. The complement component 3a receptor 1 (C3AR1) knockdown in trophoblasts disrupted Jak-STAT/TGF-β/HIF-1 signalling, reduced proliferation and invasion, and increased apoptosis, indicating that complement receptor signalling links environmental exposures and inflammation to trophoblast motility [42].

Collectively, these secondary articles reinforce the view that trophoblast invasion, differentiation, and placental/ECM remodeling function as common downstream effectors of epigenetic (lncRNAs, imprinting), endocrine (CYP11A1), and immune/complement (C3AR1) perturbations.

### 3.6. Conceptual Framework Linking the Five Mechanistic Domains

To synthesize a highly heterogeneous mechanistic literature in HDP/PE while making pathway overlap explicit, we organized findings into five recurring domains and summarized their commonly reported interconnections in Figure 2, which provides the organizing framework for Section 3 [55,56,57,58,59].

Upstream predisposition/exposures and hormonal-metabolic modulation are represented as cross-cutting modifiers, because human metabolomics-transcriptomics and endocrine receptor studies demonstrate broad effects on trophoblast behavior and downstream placental stress/vascular phenotypes [18,60,61].

Epigenetic and ncRNA regulation is retained as a distinct domain because included studies show that methylation or non-coding RNA signals can alter immune tolerance pathways and angiogenic control, positioning epigenetic regulation as a plausible upstream ‘set-point’ layer in HDP/PE biology [27,62]. For example, HLA-G promoter hypermethylation was associated with PE and linked to immune-tolerance signaling, illustrating a mechanism by which epigenetic changes may modify maternal–fetal immune adaptation and downstream placentation programs [62]. Likewise, the characterization of the MG828507 lncRNA upstream of FLT1 supports a cis-regulatory connection between ncRNA regulation and the anti-angiogenic axis, providing a direct bridge between the epigenetic/ncRNA domain and angiogenic dysregulation (Figure 2) [27].

The trophoblast invasion/differentiation and placental remodeling domain is treated separately because mechanistic studies demonstrate invasion defects and disrupted differentiation programs in trophoblast models and human placenta, which plausibly contributed to impaired placental architecture and perfusion capacity [49,63,64]. Reduced ITGB3 in preeclamptic placenta with functional effects on trophoblast behavior supports the importance of integrin-mediated adhesion and invasion programs in placental development [63]. Similarly, CLDN11 downregulation impaired trophoblast invasion and endovascular trophoblast differentiation in early-onset disease, providing mechanistic evidence consistent with incomplete spiral-artery remodeling [49].

Hypoxia-responsive migration circuitry (e.g., hypoxia-induced Notch signaling) further links placental stress to altered trophoblast motility, reinforcing the rationale for placing ‘placental stress’ as an intermediated node in Figure 2 [64].

Placental malperfusion and intermittent hypoxia-reoxygenation are positioned in Figure 2 as a central stress node consistent with observational evidence of mitochondrial abnormalities and altered biogenesis signaling in PE placenta [65].

Causal anchoring for oxidative-angiogenic coupling is supported by experiments in which mitochondrial bioenergetic modulation with AP39 reduced oxidative stress and attenuated anti-angiogenic responses in hypoxia-exposed trophoblasts [66].

Within the oxidative/redox domain, ferroptosis-related pathways provide an additional mechanistic layer because multiple studies report ferroptosis-associated signatures and lipid-peroxidation networks in PE tissues/cells or maternal leukocytes [33,34,67].

Immune, inflammatory, and complement mechanisms are considered a distinct domain because included studies demonstrate definable immune signaling pathways beyond nonspecific cytokine elevation, including sterile triggers or inflammasome activation and regulatory small-RNA control of inflammasome activity [38,45]. Sterile lipid inflammation via cholesterol crystal-associated NLRP3 signaling and miRNA-linked modulation of NLRP3 inflammasome activity illustrate reproducible immune programming at the maternal–fetal interface [38,45]. Immune activation also intersects with oxidative stress in human tissue models, as shown by placental explant experiments where hydrogen peroxide exposure influenced inflammasome expression and vitamin D modulated NLRP components, supporting cross-domain coupling represented in Figure 2 [39].

Complement dysregulation is integrated within the immune domain because complement control mechanisms (e.g., factor H) and complement receptor signaling (e.g., C3AR1) are proposed to interface with inflammatory activation and trophoblast-related signaling in PE [41,42].

Intercellular propagation routes that connect placental/immune stress to the maternal vasculature are supported by evidence that circulating extracellular vesicles and NETs contribute to endothelial dysfunction and that placental EV uptake by endothelial cells occurs via defined internalization mechanisms [29,68].

The angiogenic dysregulation/endothelial injury domain is treated as the principal vascular-effector module in Figure 2, because included studies demonstrate transcriptional regulation of anti-angiogenic mediators (including pathways that reduce sFLT1) and placental factors capable of inducing endothelial dysfunction [69,70,71]. Finally, functional vascular profiling of the PE placental circulation using combined transcriptomics and vascular tension analyses supports that measurable vascular phenotypes align with an anti-angiogenic/inflammatory milieu summarized in Figure 2 [72].

Overall, Figure 2 provides an explicit rationale for structuring the Results into five domains while recognizing that individual studies frequently span multiple domains, consistent with contemporary placental endotyping efforts [55,56,57,58,59].

## 4. Discussion

PE remains a challenge in the management of obstetrical complications and understanding the molecular and cellular mechanisms underlying this condition is essential to improving maternal and fetal outcomes [75].

This systematic review incorporates human mechanistic studies of hypertensive disorders related to pregnancy, mostly related to PE, over a 10-year window, grouped into 5 molecular mechanisms. Across this classification of the included articles, most converged on a multi-hit model in which abnormal placentation, oxidative and immunological stress, and epigenetic/ncRNA rewiring jointly lead to angiogenic imbalance and systemic endothelial dysfunction.

Most studies were judged to have ‘Some concerns’, rather than ‘Low’ or ‘High’ RoB, where the common issues were: selection of the participants (limited control for gestational age at sampling), measurement bias at the molecular level (incomplete reporting of assay validation, lack of blinding of laboratory staff), timing (sampling at delivery meant that most molecular patterns could reflect consequences or adaptative responses rather than initiating events).

### Limitations

A first limitation is the marked heterogeneity in study design, biological material, and clinical phenotypes. Most studies were small, single-centre, observational case-control or cross-sectional designs. Placental tissue dominated, but maternal plasma, serum, or leukocytes were also represented, complicating direct comparisons of effect sizes or pathway strength across mechanisms. Most samples were obtained at late gestation or delivery, with only a small subset including first-trimester or mid-pregnancy material. This means most molecular signatures reflect an established disease state rather than initial causal events.

A second key limitation is the intricate overlap between mechanisms. Many angiogenic/endothelial studies also demonstrated oxidative stress or immune activation [13,14,35]. Immune and complement studies often reported downstream effects on trophoblast behaviour, apoptosis, or syncytialization, or used circulating or placental markers that feed into the angiogenic axis [29,35].

A third limitation arises from the subset of articles for which it was difficult to identify a specific molecular mechanism. Several transcriptomic or proteomic studies produced long lists of differentially expressed features with broad pathway enrichment but did not prioritise one axis mechanistically [23,76]. A few studies focused on pathways that do not map into the five mechanisms found, without a strong connection, such as very specific receptor-ligand systems, and were not discussed in this systematic review [77,78]. Even if the included studies were analysed based on molecular mechanisms found, there is not yet a consensus to guide future interventional studies.

The limitations of current knowledge are reflected entirely in this study. Individual variability and the necessity for further research to establish causal relationships must be acknowledged. Continuing to explore these pathways in the development of PE is essential for uncovering potential therapeutic targets and prevention strategies.

## 5. Conclusions

The complex development of hypertensive pregnancy disorders reflects numerous molecular mechanisms implicated in this process. From genetic and environmental influences on placental dysfunction to epigenetic changes and systemic dysregulation, each mechanism contributes to health outcomes for both the mother and the child. Understanding these intricate relationships is critical for developing targeted interventions and public health strategies aimed at diminishing the risk of future cardiovascular complications.

## Figures and Tables

**Table 1:** Distribution of mechanistic evidence and RoB across the five molecular domains.

Mechanistic Domain	Studies Demonstrating Primary/Secondary Mechanism, *n*	Primary/Secondary, *n*	Overall RoB (Low/Some Concerns/High), *n* (%)	Primary RoB (Low/Some Concerns/High), *n* (%)	Secondary RoB (Low/Some Concerns/High), *n* (%)
Angiogenic dysregulation & endothelial injury	17	13/4	4 (23.6%)/13 (76.4%)/0 (0%)	3 (23%)/10 (77%)/0 (0%)	1 (25%)/3 (75%)/0 (0%)
Oxidative/redox imbalance & mitochondrial dysfunction	14	10/4	2 (14.2%)/12 (85.8%)/0 (0%)	2 (20%)/8 (80%)/0 (0%)	0 (0%)/4 (100%)/0 (0%)
Immune, inflammatory & complement mechanisms	13	7/6	3 (23%)/10 (77%)/0 (0%)	1 (14.2%)/6 (85.8%)/0 (0%)	2 (33.3%)/4 (66.7%)/0 (0%)
Trophoblast invasion, differentiation & placental remodeling	13	7/6	2 (15.3%)/11 (84.7%)/0 (0%)	1 (14.2%)/6 (85.8%)/0 (0%)	1 (16.6%)/5 (83.4%)/0 (0%)
Epigenetic & non-coding RNA regulation	9	5/4	2 (22.2%)/7 (77.8%)/0 (1.6%)	2 (40%)/3 (60%)/0 (0%)	0 (0%)/4 (100%)/0 (0%)

**Table 2:** Overview of study characteristics by mechanistic domain.

Mechanistic Domain	Predominant Sample Source	Predominant Timing	Common Study Designs	Typical Experimental Approaches	Reference Examples
Angiogenic/endothelial	Placenta; maternal plasma/serum	Delivery/late gestation	Case–control, cross-sectional	ELISA, IHC, qPCR; some functional endothelial assays	[13,14,15,16,17,18,19,20,21,55,57,70,71,73]
Oxidative/mitochondrial	Placenta; trophoblast models	Delivery/late gestation	Case–control	ROS assays, mitochondrial function, H/R trophoblast models	[29,30,31,32,33,34,35,58,61,65,66,67,72,74]
Immune/complement	Placenta ± maternal immune cells	Delivery/late gestation	Case–control	Cytokines, immune profiling, inflammasomes, EV/NETwork	[36,37,38,39,40,41,42,43,44,45,46]
Trophoblast/remodeling	Placenta; EVT/trophoblast models	Delivery (some early)	Case–control + mechanistic in-vitro	Invasion assays, EMT markers, MMP/TIMP, explant outgrowth	[23,25,42,47,48,49,50,51,52,53,54,63]
Epigenetic/ncRNA	Placenta; cell lines	Delivery/late gestation	Case–control	miRNA/lncRNA profiling, methylation, luciferase/rescue	[22,23,24,25,26,27,28,29,62]

Abbreviations: ELISA: Enzyme-Linked ImmunoSorbent Assay: IHC: immunohistochemistry: qPCR: quantitative polymerase chain reaction: EMT: Epithelial-Mesenchymal Transition; EV/NETwork: Extracellular vesicles network; MMP: Matrix Metalloproteinases; TIMP: Tissue Inhibitors of Metalloproteinases.

## Data Availability

No new data were created or analyzed in this study. Data sharing is not applicable to this article.

## Supplementary Materials

The following supporting information can be downloaded at: https://www.mdpi.com/article/10.3390/cimb48030294/s1.
