# Supplementary material for: New Frontiers in the Pathophysiology of Hypertensive Pregnancy Disorders: A Systematic Review of Molecular Insights into Preeclampsia
Source: Curr Issues Mol Biol. 2026 Mar 10;48(3):294. doi: 10.3390/cimb48030294 (PMC13025347; doi:10.3390/cimb48030294)
Supplement: Supplementary file 1 [file cimb-48-00294-s001.zip › Supplementary File S1.pdf]

New Frontiers in the Pathophysiology of Hypertensive Pregnancy Disorders: A Systematic Review of Molecular Insights into Preeclampsia

-Search Strategies-

This appendix records the exact queries used in each database, together with filters and the search date, for PRISMA reproducibility.

1. PubMed

Final Query Used	(("Preeclampsia"[Mesh] OR "Eclampsia"[Mesh] OR "Hypertension, Pregnancy-Induced"[Mesh] OR preeclampsia[Title/Abstract] OR eclampsia[Title/Abstract] OR "gestational hypertension"[Title/Abstract] OR "hypertensive disorders of pregnancy"[Title/Abstract]) AND ("molecular mechanism"[Title/Abstract] OR "signal transduction"[Mesh] OR "gene expression"[Mesh] OR "oxidative stress"[Mesh] OR epigenetic[Title/Abstract] OR "placental dysfunction"[Title/Abstract] OR "pathogenesis"[Title/Abstract] OR "pathway"[Title/Abstract])) AND (Humans[Mesh]) AND (English[lang]) AND ("2015/01/01"[Date - Publication] : "2025/09/01"[Date - Publication]) AND (Clinical Trial[ptyp] OR Observational Study[ptyp] OR Randomized Controlled Trial[ptyp]) AND (hasabstract[text])
Filters Applied	Year: 2015–2025; Language: English; Species: Humans; Article types: Clinical Trial, Randomized Controlled Trial, Observational Study; Full text: Yes

Date Searched	searched on Oct 18, 2025

2. Scopus (Advanced Search)

Final Query Used	TITLE-ABS-KEY (preeclampsia AND ("molecular mechanism" OR "oxidative stress" OR "gene expression" OR epigenetic) AND placenta) AND PUBYEAR > 2014 AND PUBYEAR < 2026 AND (LIMIT-TO (SUBJAREA , "MEDI")) AND (LIMIT-TO (DOCTYPE , "ar")) AND (LIMIT-TO (PUBSTAGE , "final")) AND (LIMIT-TO (SRCTYPE , "j")) AND (LIMIT-TO (LANGUAGE , "English")) AND (LIMIT-TO (OA , "all"))
Filters Applied	Year: 2015–2025; Language: English; Source type: Journals; Subject area: MEDI; Doc type: Article; Publication stage: final; Open Access: all
Date Searched	searched on Oct 18, 2025

3. ScienceDirect (Main search + filters)

Final Query Used	"gestational hypertension" AND ("molecular mechanism" OR epigenetic) AND placenta AND pregnant
Filters Applied	Year: 2015–2025; Language: English; Article type: Research articles; Full text: Yes; Subject areas: Medicine, Biochemistry, Molecular Biology, Obstetrics & Gynecology

Date Searched	searched on Oct 18, 2025
